# Entropy Profiles for Li-Ion Batteries—Effects of Chemistries and Degradation

**DOI:** 10.3390/e27040364

**Published:** 2025-03-29

**Authors:** Julia Wind, Preben J. S. Vie

**Affiliations:** Institute for Energy Technology (IFE), NO-2007 Kjeller, Norway; julia.wind@ife.no

**Keywords:** Li-ion battery, entropy, reversible heat, thermal characterization

## Abstract

This paper presents entropy measurements for a large set of commercial Li-ion cells. We present entropy data on full cells with a variety of common Li-ion cell electrode chemistries; graphite, hard carbon, lithium-titanium-oxide (LTO), lithium cobalt-oxide (LCO), nickel manganese cobalt oxides (NMC), nickel cobalt aluminium oxide (NCA), lithium iron-phosphate (LFP), as well as electrodes with mixes of these. All data were collected using an accelerated potentiometric method in steps of approximately 5% State-of-Charge (SoC) across the full SoC window. We observe that the entropy profiles depend on the chemistry of the Li-ion cells, but that they also vary between different commercial cells with the same chemistry. Entropy contributions are quantified with respect to both, their means, positive and negative contributions as well as their SoC variation. In addition, we present how different cyclic ageing temperatures change the entropy profiles for a selected commercial Li-ion cell through ageing. A clear difference in entropy profiles is observed after a capacity loss of 20%. This difference can be attributed to different ageing mechanisms within the Li-ion cells, leading to changes in the balancing of electrodes, as well as changes in the electrode materials.

## 1. Introduction

Li-ion batteries (LIBs) have emerged as the current major commercial rechargeable battery technology. Since its commercial launch in the 1990s Li-ion batteries have been further developed and steadily improved. Initially, the LIB replaced other secondary batteries as e.g., NiMH in portable electronics (e.g., mobile phones and portable computers), followed by the introduction of LIBs into electric vehicles (EVs) through the Tesla Roadster in 2008, the Mitsubishi i-MiEV and Nissan Leaf in 2010. Since 2010, the LIB has also been used in electric boats, with the first fully electric car ferry starting operation in 2014 [1] and for large-scale electric energy storage [2].

The LIB is known for its very high energy and power density as well as a superior round-cycle efficiency of commonly more than 95%. Currently, there are no other commercial secondary battery technologies with a higher energy density than LIBs. Possibly, the LIB can in future be replaced by the all-solid-state battery (ASSB) based on Li-metal as one electrode with prospects of even higher energy densities. However, this technology is not yet commercially available and will most likely have a much higher initial cost.

Due to its high energy density, as well as consisting of flammable materials (e.g., graphite electrode, polymer separator and electrolyte solvents), the LIB is known to have challenges with its safety properties. Among the most well-known and previously reported safety incidents and issues are the ones reported by the Boeing 787 Dreamliner (2013) [3] and Samsung Galaxy Note 7 (2016) [4]. These issues were caused by internal faults within the battery cells, as well as issues with the engineering of the overall battery system (Boeing). More recently, reports of fires in “e-scooters” during charging have become a recurring issue [5]. This is quite often linked to the use of low-quality cells in relatively cheap products. There are also several reports of fires in EVs, but the reported number of fires are not higher in EVs compared to fires in normal cars with internal combustion engines. There have also been reported fires in battery-based propulsion systems in ships [6,7,8] as well as several fires in large stationary energy storage systems [9,10]—all based on LIBs. There are several causes for these fires. Some were caused by a misfitted gasket in the battery liquid cooling system causing a short-circuit of the battery system [6,8], another had a design flaw where saltwater could reverse through the ventilation system and again short-circuit the battery system [7] while others again were caused by internal cell faults or had unknown causes [10].

The major causes of fires in Li-ion-based battery systems can hence be attributed to external factors like e.g., external short-circuits or overheating that may lead to overheating and eventually force the battery into a thermal runaway event. However, fires in LIBs can also arise from internal faults within the battery cell. LIBs can have faults from production that can cause internal short circuits, while battery ageing and abuse may also cause safety-critical events. One well-known factor that can lead to an internal short is lithium plating that can occur during the charging of LIBs at relatively low temperatures and relatively high currents [11,12,13,14,15,16]. During lithium-plating, the lithium ions are not able to access the graphite sites for intercalation at a high enough rate and will instead be deposited on the surface of the graphite as metallic lithium. The metallic lithium can then grow in a dendritic manner that can potentially penetrate the separator between the anode and cathode and eventually short-circuit the battery from within through a so-called “local short” or “weak short”. This could potentially lead to a full fire of the LIB but could also only cause a local short circuit that damages the battery in a smaller localized area. Upon the possible detection or diagnosis of an onset of lithium plating, a major safety risk in LIBs could be mitigated.

During lithium plating, there is an irreversible loss of cyclable lithium within the battery on the graphite anode. This process is commonly known as loss of lithium inventory (LLI) and is one of several known ageing modes in LIBs [17]. Another process leading to LLI is the buildup of the SEI layer (see e.g., [18]) on the anode surface. The two other ageing modes are the loss of active material (LAM), and impedance increase due to reaction kinetics degradation [17]. The detection and identification of rapidly increasing LLI could imply that the LIB is going through a possible hazardous plating of lithium metal on the graphite electrode. The LLI implies a shift in balance between the anode and cathode electrodes and can hence be detected by several different methods. These methods entail e.g., the diagnostic techniques of DVA and ICA (see e.g., [17,19,20]). It is however outside the scope of this paper to elaborate more on these specific techniques. We will in this paper show that changes to the entropy profile through the course of ageing can be used as another technique to reveal LLI, and hence possibly lithium metal plating.

Detailed knowledge of the heat development from a LIB can be crucial for the detection of a sudden safety event, as well as provide a full understanding of the local temperature profile during both charge and discharge. It is well known that the developed heat from a LIB comes from both reversible and irreversible heat sources [21,22]. The irreversible heat is linked to the internal resistance and overpotentials and will always generate heat, while the reversible heat can contribute to both heating and cooling of the battery. The reversible heat stems from the change in entropy in the two electrodes of the LIB and will vary with the Li-ion concentration and thus the battery’s state of charge (SoC). Some cooling effects of the entropy can be observed in most LIB temperature profiles. This can be seen, e.g., in Figure 8 in [23] where the large variations in the temperature profiles of both charge and discharge are caused by the variations in the LIB’s entropy profile. The lowered temperatures in certain parts of the SoC window illustrate this cooling effect. During discharge, a positive entropy value will contribute to cooling the battery cell, while during charge a negative entropy will do the same. The entropic heat may even balance out or be larger than the irreversible heat contributions caused by, e.g., the internal resistance. The change in entropy is caused by phase transitions in the electrode materials [24,25,26]. A shift in a cell’s temperature profile and hence the entropy profile, can reveal a shift in the electrode balancing of the cell. This in turn could be used as another tool to detect critical LLI in a cell, possibly caused by lithium metal plating [27].

The entropy of the LIB at any SoC can be measured by several methods, e.g., the potentiometric and calorimetric methods. In the potentiometric method, the change in open circuit voltage (OCV) is measured upon a corresponding change in cell temperature [25,28]. The change in entropy is then given by $∆S=nF\frac{{\partial U}_{OCV}}{\partial T}$, where $\frac{{\partial U}_{OCV}}{\partial T}$ is the derivative of the OCV ($U_{OCV})$ with respect to temperature (T). The calorimetric method involves measuring the heat from the Li-ion cell during charge and discharge where different models are applied to differentiate between irreversible and reversible heat sources [29,30,31,32,33].

This paper presents the measurements of entropy profiles for a large range of LIBs. A total of 14 different cells were measured using the accelerated potentiometric method [23]. The cells had different anode and cathode chemistries as well as different geometries (i.e., pouch, cylindrical or prismatic) and capacities ranging from 0.4 to 64 Ah. All cells were measured at the beginning of life. In addition, the entropy profile of one specific cell was followed through ageing at different temperatures.

The paper is structured as follows: we first introduce the procedure for the measurement of the entropy profiles (Section 2.1), and present an overview of the tested batteries (Section 2.2). The measured entropy profiles are presented and discussed with respect to electrode chemistries (Section 3.1), and quantified with respect to means, standard deviations as well as positive and negative contributions in Section 3.2. A comparison between entropy profiles at charge and discharge is presented in Section 3.3. Finally, the evolution of an entropy profile for a cell through ageing is presented (Section 3.4) before the results are discussed (Section 4).

## 2. Experimental/Materials and Methods

### 2.1. Method for Entropy Measurements

The method applied for obtaining entropy profiles was an accelerated potentiometric method discussed in detail in Hua [23]. The entropy was measured in 5% SoC steps across the full SoC window with an accelerated potentiometric method for both charge and discharge of the cell. The method and apparatus were previously described in full [23]. The LIBs were placed in thick-walled plastic bags before immersion into a high-precision water bath. This enabled good thermal contact and a stable cell temperature. The protocol used for measuring the entropy for both discharge and charge was the following [23]:

1. Charge the cell to the maximum voltage limit for the cell (100% SoC) (CC-CV charge with a 0.05C current cut-off).
2. Allow cell voltage (OCV) to stabilize to a relaxation rate lower than 1.4 mV/h or a maximum relaxation time of 6 h.
3. Cycle the temperature of the water bath from 25 °C to 16 °C, and back to 25 °C via steps of 3 °C increments from 16 °C. At every temperature step, the OCV and temperature were allowed to stabilize before the next temperature step. The temperature was assumed stable when the standard deviation of a five-minute time window was smaller than 0.01 °C. The OCV of the cell was recorded continuously.
4. The SoC was changed in steps of approximately 5% through a constant 0.05C current (CC) discharge or charge. (The cell will in steps of 5% SoC eventually reach the lower voltage limit (0% SoC) and then be charged back to 100% SoC in 5% SoC steps)
5. Loop back to step 2.

The respective maximum and minimum voltage limits for the different cells are presented in Table 1.

For two of the cells (LG JP3 and LG E58C), the measurements were conducted in a slightly different way: instead of the equipment presented in [23], a conventional battery tester (PEC ACT-0550) was used to change SoC and record OCV and temperature. The cell temperature was controlled by a programmable incubator (VWR INCU-Line 150R) and only two different temperatures (25 and 15 °C) were used to measure the change in OCV following the temperature change.

### 2.2. The Tested Cells

A total of 14 different LIB cells were investigated in this study over the course of approximately 10 years. The set is comprised of cells of a large variety of different chemistries, form factors, energy densities and manufacturers. Cell chemistries include standard graphite, LTO as well as hard carbon on the anode side, combined with cathode chemistries of LCO, different types of NMC, LFP, NCA as well as a blend of NMC532/LCO/LMO. Form factors include pouch, prismatic as well as cylindrical cells, with capacities from very small 0.36 Ah (cylindrical) and up to 64 Ah (pouch). An overview of all cells and relevant details are summarized in Table 1.

**Table 1:** Overview of the cells measured for full cell entropy and their properties. A separate column gives references to previous literature that has been published by the researchers in the group on the same cell type. The entropy data for the Xalt 31 HE [34] and Melasta cells [35] was published in previous works and is included here for completeness.

Name	Manufacturer & Cell Model	Capacity [Ah]	Voltage Range [V]	Form Factor	Chemistry	Energy Density [Wh/kg]	Comments/ References
Kokam LTO	Kokam SLPB65205130N	11	1.5–2.8	pouch	LMO(?)- LTO	69	Cathode chemistry unconfirmed
Toshiba SciB	Toshiba SciB 20	20	1.5–2.7	prismatic	LMO(?)/- LTO	89	Cathode possibly LMO [36,37]
LG JP3	LGV JP3	64	3.0–4.2	pouch	NMC532- graphite	210	[38,39]
LG E58C	LG E58C	58	3.0–4.2	pouch	NMC622- graphite	242	Cell harvested from a Jaguar I-pace battery
Xalt 31 HE	Xalt 31 HE	31	2.7–4.2	pouch	NMC111- graphite	180	[34,38]
Panasonic UR	Panasonic UR18650W	1.5	2.75–4.2	cylinder	NMC- graphite	120	[40,41]
Enerdel	Enerdel 151105203A 102401	17.5	2.5–4.1	pouch	NMC- hard carbon	147	[42]
Panasonic NCR	Panasonic NCR18650B	3.25	2.5–4.2	cylinder	NCA- graphite	243	[41]
SAFT VL6P	SAFT VL6P	6	2.7–4.0	cylinder	NCA- graphite	74	[40]
SAFT VL30PFe	SAFT VL30PFe	30	2.5–3.8	cylinder	LFP- graphite	91	[40]
Melasta 8C	LPB042126-8C	7	3.0–42	pouch	LCO- graphite	190	[35,39]
Melasta 10C	SLPBB042126-10C	6.55	3.0–42	pouch	LCO- graphite	188	[35,39]
Samsung SDI	ICP103450A	2	2.75–4.2	prismatic	LCO- graphite	-	
GPB ICR10440	Great Power Battery Co Ltd. ICR10440	0.36	3.0–4.2	cylinder	NMC532/ LCO/LMO-graphite	69	[43]

### 2.3. The Cycle Life Study

In addition to entropy measurements performed at the cells’ beginning of life, the entropy profiles of the Xalt 31 HE cell were studied while the cell was aged as part of a cycle life study. The corresponding ageing results for this cell were previously published [38], the entropy profile at 100% SoH (State-of-Health) (100% State-of-Health (SoH) refers to a cell with no loss in cell capacity) was used to exemplify the new methodology presented [23].

In this work, we present ageing results of cells cycled in the full voltage window from 2.7 V to 4.2 V at a constant current of 1C, at three different temperatures of 5, 25 and 45 °C. All charging of the cells was performed with a constant current charge to the maximum voltage and finished with a constant voltage hold until the current was reduced to less than 0.1C. The cells were tested without mechanical compression and were allowed to cool down to at least 1 °C above the set temperature before starting a new charge or discharge during cycling with otherwise a minimum OCV period of 5 min. The cells were all characterised at 25 °C at regular intervals of a maximum of 200 cycles (The term “cycles” refers to total absolute cycles in this algorithm), or earlier when a cell lost 5% capacity during the cycling. The characterisation test included two cycles of testing capacity at both 0.1C and 1C in the full SoC window. The results presented here were part of a larger ageing study where the effects of different temperatures, currents, and voltage limits were scrutinised and have partly been published [38].

### 2.4. Data Handling

For the direct comparison of the measured entropy profiles and their statistical analysis, the data was treated in the following way before calculating means and standard deviations: entropy data points were resampled to *n* = 200 equidistant points across the SoC window, interpolated (1d cubic) and smoothed (Gaussian smoothing, sigma = 2). Integrals were calculated using the trapezoidal method. All data handling was conducted in Python, using standard methods as implemented in the scipy (v1.13.0) package.

## 3. Results and Discussion

**Figure 1:**
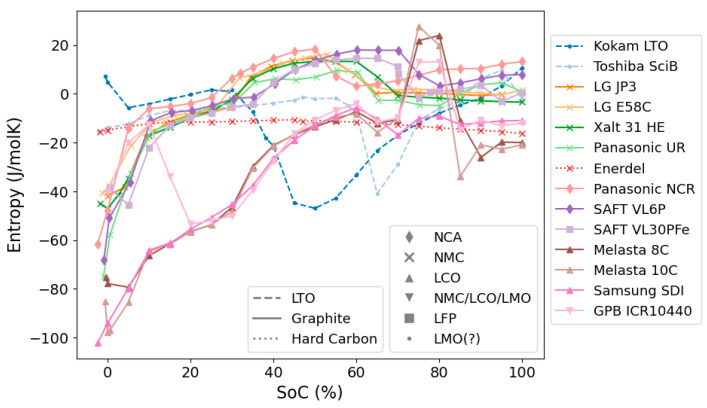
Overview of measured discharge entropy profiles for all cells. Different anode and cathode chemistries are represented by line styles and marker symbols, respectively.

An overview of the measured entropy profiles of all the investigated cells at 100% SoH is presented in Figure 1. The respective anode and cathode chemistries for the individual cells are highlighted (for all cell details see Table 1 above). All profiles in the figure were obtained during discharge of the battery. In the following sections, we look at and discuss the similarities and differences of these profiles in more detail.

### 3.1. Differences in Entropy Profiles by Electrode Chemistry

**Figure 2:**
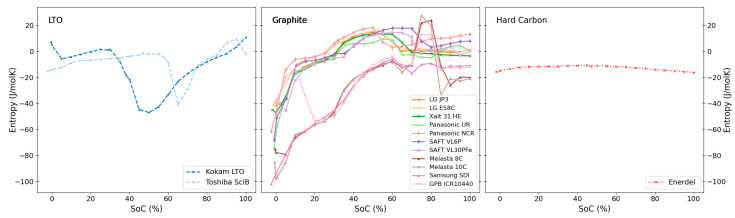
The entropy profiles of all tested full cells sorted by the known anode materials; LTO, graphite or hard carbon.

**Figure 3:**
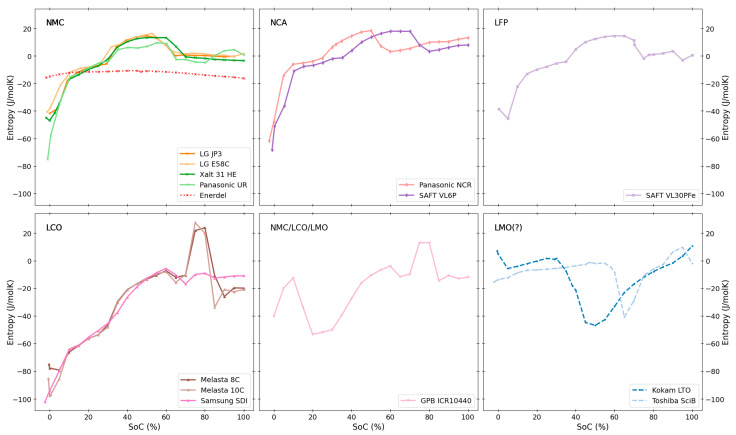
The entropy profiles of all tested full cells sorted by the known cathode materials; NMC, NCA, LFP, LCO, NMC/LCO/LMO (blend) and two cells with likely (but unconfirmed) LMO cathodes.

Figure 2 and Figure 3 show the entropy profiles grouped by anode and cathode chemistries. Similarities and differences across the two figures allow for the identification and confirmation of the individual electrode chemistries’ contribution to the entropy variation of the full cell.

*Hard Carbon.* One of the investigated cells (Enerdel) contained a hard-carbon anode, combined with an NMC cathode. For this cell, we observe a profile with almost constant entropy across the entire SoC window (see Figure 2: Hard Carbon). The entropy values across the full SoC window are all within the range of −16.3 to −10.6 J/molK, with a SoC-averaged entropy value of 13 J/molK. This indicates that both electrode active materials, hard carbon and NMC, have little variation in entropy with a change in SoC. For hard carbon specifically, this can be rationalised by the material’s almost amorphous structure. The influence of structural disorder on the thermodynamics of lithium intercalation into graphite and disordered carbons was discussed in detail by Reynier et al. [44]. In their work, they point out a clear correlation between the magnitude and variation of the entropy and the state of order in the carbon. In contrast to the characteristic features in the entropy profiles observed for graphite electrodes (see next paragraph), only small entropy values and entropy variations were observed for more disordered carbons. These observations also agree with the findings reported by Takano et al. [25] for an LCO/hard carbon cell: Takano et al. [25] attributed the main contribution to the cell’s entropy profile to the LCO cathode, with negligible contributions from the hard-carbon anode.

*Graphite.* Cells with graphite anodes exhibit large similarities in their entropy profiles across the range of different investigated cathode chemistries, NMC, NCA, and LFP. The most prominent features in these entropy profiles arise from the different defined intercalation stages of Li into graphite. These intercalation stages are commonly labelled by the number of graphite layers between layers of intercalated lithium and are well established and described in detail in literature (see e.g., [45]). The entropy profiles (see Figure 2: Graphite) show large negative values and a rapid increase at low SoCs (dilute stage 1L), levelling off at around 10% SoC (formation of stage 4). A prominent broad peak is then observed between 40–60% SoC, corresponding to the formation of stage 2 graphite. As all presented profiles arise from full cells, details on the entropy profiles of course also depend on the respective cathode chemistries. Especially NMC (as observed above when paired with hard carbon), LFP and NCA cathode chemistries seem to only contribute very little to the overall entropy profile (see separate discussions on cathode chemistries below). Consequently, the main contribution to these profiles can be attributed to the graphite anodes. The main differences between these profiles are the exact positions of the steep change in entropy at low SoC as well as the position and width of the prominent broad peak (stage 2 formation). These variations are attributed to differences in the balancing between anode and cathode in the respective cells. These profiles are in agreement with earlier literature reports on an NMC/graphite full cell [46], and align with numerous reports on entropy coefficients for graphite half cells (see e.g., summary provided by Gunnarshaug et al. [47] and references therein [24,26,44]). Pairing graphite with LCO-containing cathodes significantly influences the overall entropy profiles [25], leading to overall significantly larger negative entropy values, in addition to the significant LCO peak at around 80% SoC. This will be discussed in more detail in the LCO section below.

*LTO.* Both LTO-based cells have quite similar distinct entropy profiles that are very different compared to the ones for graphite-based cells. The full LTO profiles show some similarities to the cell with a cathode blend (NMC/LCO/LMO, see Figure 3). The “valley” at around 25% SoC for the cathode blend resembles a similar “valley” at slightly higher SoCs for the LTO cells. The LTO cells’ entropy profile is likely largely influenced by the cathode chemistry, hinting towards the cathode chemistry being LMO-based, as indicated by Appiah [36] and Koshtyal et al. [37]. The different SoC windows for the valley can stem from different balancing of the electrodes which is natural due to the substantially different anode chemistries (LTO vs. graphite). Entropy profiles for LTO half-cells are reported to show very low magnitude (around −5 J/molK) and little SoC variation [48,49]. Overall, LTO-based anodes contribute little to a cell’s total entropy profile, in agreement with the LTO profiles presented by Viswanathan [26].

*LCO.* The profiles for LCO-containing cells (see Figure 3: LCO) are characterised by large negative entropy values across the majority of the SoC window. The almost linear increase in entropy up to around 70% SoC (i.e., *x* = 0.6 to *x* = 0.8 in Li*_x_*CO_2_) arises from the Li intercalation into the hexagonal LCO phase [44]. This is followed by a peak with positive entropy at around 70–90% SoC. The pronounced peak is commonly attributed to the hexagonal to monoclinic phase transition (around *x* = 0.55 in Li*_x_*CO_2_). The monoclinic phase is known to have a significantly higher entropy compared to the hexagonal phase [44]. In our work, all three cells with LCO cathode chemistries show very similar entropy profiles in agreement with literature [26,44], except for the area around 80% SoC in which the two Melasta cells show the pronounced peak that the third cell (Samsung SDI) is lacking. This peak is also the main differentiating feature in the direct comparison of entropy profiles for different types of LCO cells by Viswanathan [26]. However, no further reasoning for this was given. The hexagonal to monoclinic phase transition and consequently the intensity of the peak at 80% SoC was reported to be highly dependent on temperature and the presence of small amounts of different elements (such as e.g., Ni) or excess lithium in the cathode material [25]. This could thus explain the observed difference in this area between the different LCO cells. However, to further pinpoint the causes for the differences between the LCO cells, more detailed experiments, such as e.g., the structural characterisation of the respective active materials would be necessary. In general, LCO cathodes exhibit large overall absolute contributions to the entropy profile (especially compared to other positive cathode chemistries such as NMC, LFP, or NCA) [26,44,50].

*NMC*. As already indicated by the NMC/hard carbon and NMC/graphite profiles, NMC seems to contribute very little to the overall entropy profile of the cell. This agrees with literature reports [26,46,51,52,53] and indicates that NMC is thermodynamically more stable compared to LCO. As discussed above, the measured NMC/graphite profiles all closely resemble each other and are dominated by the characteristic graphite features. Minor differences can be attributed to changes in electrode balancing, or different transition metal ratios. Sturm et al. [52] hint towards slightly higher entropy values especially at low lithiation degrees for high-Ni NMC (NMC811) as compared to e.g., NMC111. However, these differences are small in relation to the entropy contribution from the graphite counter electrode. Here we observe slightly higher entropy within the lower SoC range for the NMC with the highest Ni content (LG E58C with NMC622), and lower entropy values for the Panasonic cell with unknown Ni:Mn:Co ratios above ~35% SoC. The subtlety of these differences does not allow for any further conclusions from the available full-cell data. A systematic investigation of the half-cell entropies for different Ni:Mn:Co ratios, comparable to the one presented by Schlueter et al. [54] for LMO cathodes, could bring valuable insights to this topic and is suggested for future work.

*LFP*. The obtained entropy profile for the LFP/graphite cell agrees well with the ones reported in literature [55,56]. The main contribution is, again, attributed to the graphite electrode with very small, almost SoC-independent contributions from the LFP cathode. Entropy profiles for LFP cathodes (half-cell data) were shown to have low magnitudes and only subtle variations with SoC, varying between −6 J/molK at low SoC to around −3 J/molK at higher SoC [56].

*NCA*. Similarly to NMC and LFP cathode chemistries, NCA is also observed to have only minor contributions to the overall entropy profile upon pairing with graphite, in agreement with literature reports [57,58].

*NMC/LMO/LCO (and LMO)*. The blended-cathode cell resembles selected parts of several entropy profiles of other cells. Visually we observe a distinct similarity with the pure LCO-based cells at higher SoC (cf. peak at 80% SoC). However, at lower SoC, the profile is more similar to the LTO-based cells, which we found indications of having an LMO cathode. LMO cathodes were also reported to show small overall entropy with a characteristic SoC variation dependent on Li:Mn ratios [26,54,59]: Across most Li:Mn ratios, the main features are a positive peak with its maximum around *x* = 0.65 (*x* in Li_x_Mn_2_O_4_), followed by a valley around *x* = 0.55, both related to transitions from disordered solid solutions to an ordered phase at *x* = 0.5 [54]. The intensity and exact location of these minima and maxima strongly depend on the exact Li:Mn ratios as well as the balancing of the electrodes within the respective full cells.

The discussed characteristic entropy features by electrode chemistry and references to previous literature reports are summarised in Table 2.

### 3.2. Quantitative Comparison of Entropy Variation

**Figure 4:**
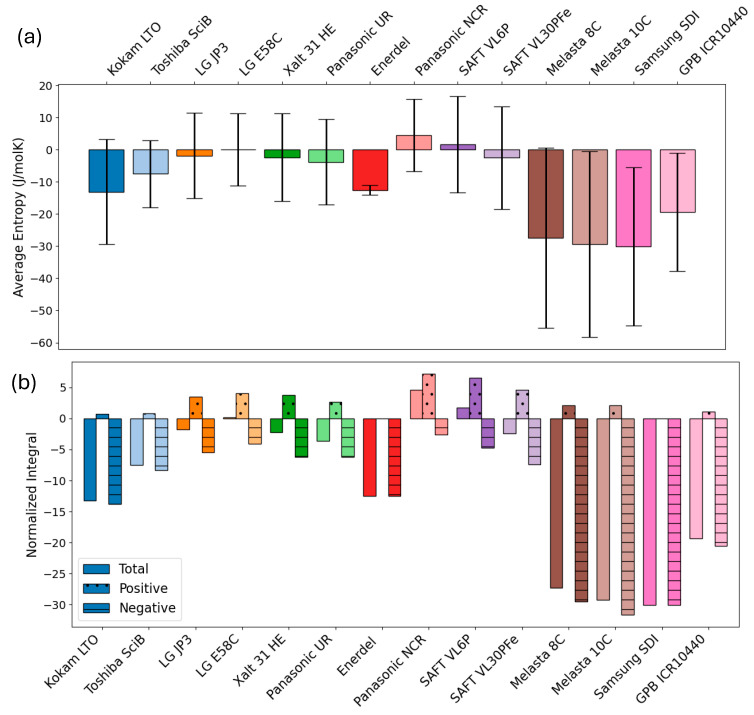
(**a**): Mean entropy values across the entire SoC window including standard deviation during discharge. (**b**): Normalized integrals (J/molK) over the full SoC window for the measured discharge entropy values and their respective positive and negative contributions for all measured cells (cf. Table 3). Colours match the overview plots in Figure 1, Figure 2 and Figure 3.

Figure 4a summarises and directly compares the SoC-averaged entropy values for all the measured cells. The entropy variation across the entire SoC window is quantified by the standard deviation and illustrated by the added error bars in Figure 4a. Note that for increased comparability, the obtained entropy data was resampled to the same number of equidistant points across the SoC window for the calculation of both means and standard deviations, as described in the methodology section (Section 2.4).

LCO-based cells clearly exhibit the largest overall negative entropy values. For cells with graphite anodes that are paired with cathode chemistries with moderate entropy changes, such as NMC, NCA, and LFP, the entropy across the entire SoC window almost averages out to zero. All cell chemistries, except for NMC/hard carbon (Enerdel), show a significant variation of the entropy with SoC, illustrated by the large standard deviation included in Figure 4a. While providing a quick and simple overview, these average values and standard deviations across the entire SoC window only give very limited insights into potential heating and cooling needs specific to the prevalent battery chemistry.

In Figure 4b we present the split of the overall average entropy into its positive and negative contributions. For this, we make use of normalised integrals across the entire SoC. The normalised integral values are directly related to the entropic heat generated (negative) or consumed (positive) during a full discharge from 100% to 0% SoC. If the total integral value is negative, there is a net entropic heat release during discharge, while there is a net entropic heat consumption if the total integral is positive. The split into negative and positive contributions illustrates the additional heat generated and consumed during battery discharge across the full SoC window (see, e.g., temperature profile during discharge in Figure 8 [23]).

**Figure 5:**
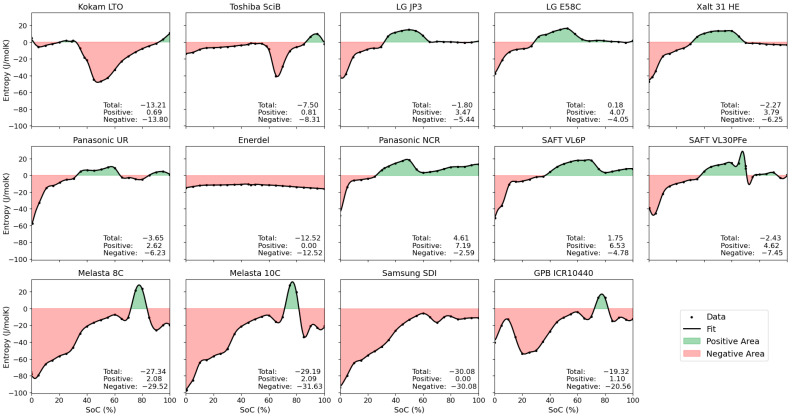
Illustration of the split of positive (green areas, cooling effect upon discharge) and negative (red areas, heating effect upon discharge) contributions to the overall entropy of the cells. The stated values are the obtained normalised integrals across (i) the entire SoC window, (ii) SoC areas with positive and (iii) negative entropy values. All units are J/molK.

For even more detailed insights, Figure 5 illustrates positive and negative contributions to the overall entropy and their SoC dependence. This overview clearly highlights the relevant SoC ranges for entropy-related heating and cooling effects. LCO-based cells show negative entropy contributions across the majority of the SoC window, with positive contributions only located at around 80% SoC. NMC, LFP, and NCA cathodes have very moderate absolute entropy changes compared to other cathode materials such as LCO or LMO, in agreement with [56]. For these materials, graphite constitutes for the main contribution and SoC variation, with significant negative entropy contributions at lower SoCs (<30%), positive contributions between around 30–70% SoC, and rather small entropy contributions at higher SoCs. This leads to an overall small average entropy in the full cells. Note here that the exact SoC values of course depend on the balancing of the electrodes in the respective cells.

The obtained values for normalised integrals, the respective positive and negative contributions as well as means and standard deviations are compiled in Table 3. Note that differences between the sum of positive and negative integrals and the overall integral is due to numerical errors.

**Table 3:** Tabulated values for normalised integrals, means, and standard deviations (* Integrals refer to normalised integrals and are calculated on resampled, interpolated and smoothed data; ** mean entropy and standard deviation (stdv) calculated from resampled values across SoC 0–100%.) All units are J/molK.

Cell Label	Chemistry	Integral *	Positive Integral *	Negative Integral *	Mean **	Stdv **
Kokam LTO	LMO(?)- LTO	−13.21	0.69	−13.80	−13	16
Toshiba SciB	LMO(?)- LTO	−7.50	0.81	−8.31	−7	11
LG JP3	NMC532- graphite	−1.80	3.47	−5.44	−2	13
LG E58C	NMC622- graphite	0.18	4.07	−4.05	0	11
Xalt 31 HE	NMC111- graphite	−2.27	3.79	−6.25	−2	14
Panasonic UR	NMC- graphite	−3.65	2.62	−6.23	−4	13
Enerdel	NMC- hard carbon	−12.52	0	−12.52	−13	2
Panasonic NCR	NCA- graphite	4.61	7.19	−2.59	5	11
SAFT VL6P	NCA- graphite	1.75	6.53	−4.78	2	15
SAFT VL30PFe	LFP- graphite	−2.43	4.62	−7.45	−3	16
Melasta 8C	LCO- graphite	−27.34	2.08	−29.52	−27	28
Melasta 10C	LCO- graphite	−29.19	2.09	−31.63	−29	29
Samsung SDI	LCO- graphite	−30.08	0	−30.08	−30	25
GPB ICR10440	NMC532/ LCO/LMO-graphite	−19.32	1.10	−20.56	−19	19

### 3.3. Difference in Charge and Discharge Entropy

**Figure 6:**
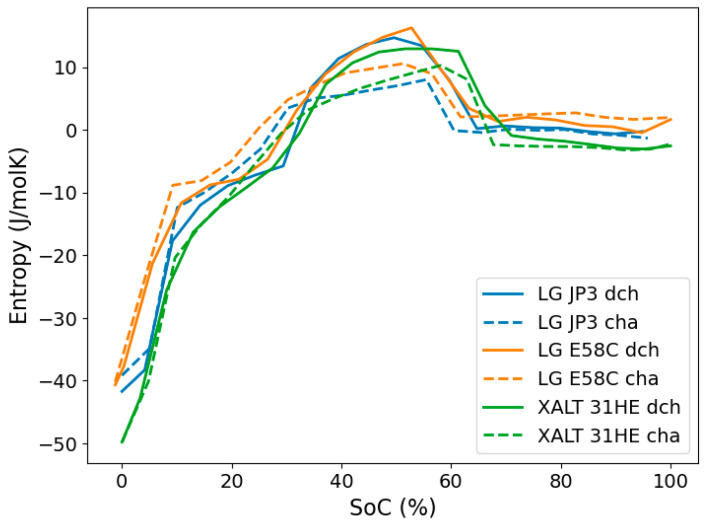
Direct comparison of charge (dashed lines) and corresponding discharge (solid lines) entropies for three selected cells.

Both charge and discharge entropies were measured for three selected cells, the two LG cells as well as the XALT cell, all with NMC/graphite chemistry. The obtained profiles are shown in Figure 6. A similar hysteresis effect between charge and discharge entropy was observed for all three cells. The charge entropy profiles are shifted to slightly lower SoCs, with an increasing accumulating hysteresis effect with increasing SoC. Hysteresis typically refers to OCV hysteresis, the remaining difference between the charge and discharge voltage curves of a battery when approaching very small currents or long relaxation times (see e.g., [62,63,64]). OCV hysteresis also leads to entropy hysteresis, i.e., the difference in entropy variation with SoC based on whether the data was collected during charging or discharge [58]. Hysteresis in entropy data was reported in several literature studies (e.g., [55,61,65]). However, there is still no clear consensus in the literature on the “true” origins of entropy hysteresis. Schmid et al. attribute the hysteresis behaviour to the potentiometric measurement methodology, speculating that a calorimetric approach should result in identical charge and discharge entropies. Similarly, Bedürftig et al. [60,61] were not able to fully clarify the origins of entropy hysteresis, also pointing towards the need for further research for more definite conclusions.

With respect to the magnitudes of the measured entropy profiles, neither charge nor discharge profiles are consistently shifted to lower or higher values with respect to each other. This observation is in perfect agreement with the reported entropy profiles for charge and discharge for the LG JP3 cell presented by Reiter [60], and other literature reports such as e.g., [60,61]. However, it is worth noting, that the absolute values for charge entropy coefficients for all measured cells in this work are almost consistently lower than the discharge entropy coefficients across the entire SoC window. This indicates slightly lower contributions of entropic heat during the charging process.

### 3.4. Ageing & Degradation

**Figure 7:**
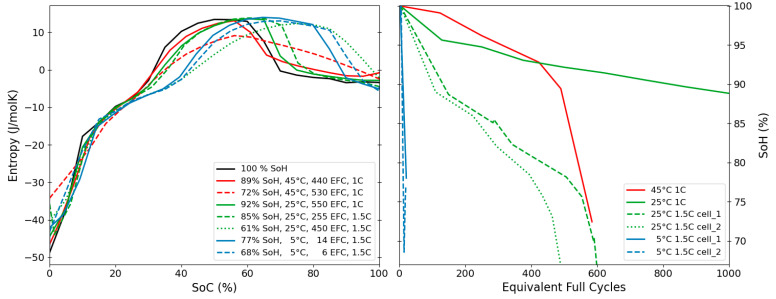
Left: Evolution of entropy changes for the XALT cell upon ageing at different temperatures and C-rates. Right: Corresponding lifetime data.

For the XALT cells (NMC/graphite), the evolution of the entropy profiles was followed throughout the lifetime of the cell when cycled at different temperatures, 5, 25 and 45 °C. Entropy profiles at selected SoHs and the corresponding lifetime data are shown in Figure 7. For all conditions, the most pronounced change is the shift of the entropy profiles towards higher SoCs, often accompanied by a horizontal stretch, with the largest shift caused by cycling at 5 °C. While cycling at low and intermediate temperatures largely preserved the main characteristic features and maximum observed entropy values, cyclic ageing at 45 °C led to a significant broadening and flattening of the main features.

As reported in Spitthoff et al. [38], the cells show very poor cycling performance at 5 °C even at 1C. The two cells within this study were cycled at an even higher C-rate of 1.5C and exhibited a very steep decrease in SoH, dropping down to 77% SoH after only 6 equivalent full cycles (EFCs) for cell 1 and 68% SoC after 14 EFCs for cell 2 respectively. The corresponding resistance increase was reported to be very low, likely owing to the low number of undergone cycles. The prevalent degradation mechanism was assumed to be irreversible lithium plating, leading to a significant loss of lithium inventory (LLI), in agreement with the observed shift and stretch of the entropy profile indicative of LLI [57]. The otherwise almost unchanged entropy signature of the cell indicates that the structural integrity of the graphite and thus the prevalent intercalation mechanisms are largely preserved.

At higher temperatures, loss of active material (LAM) on both electrodes was identified as the main cause of degradation. This can be related to structural changes/degradation of the materials and thus explain the significant change in the shape of the entropy profile at low SoH that is most pronounced for the cells cycled at 45 °C/1C and 25 °C/1.5C respectively. Inhomogeneous degradation is likely to further exaggerate this effect.

This example clearly illustrates that ageing under different conditions can have a significant influence on the entropy profiles of a battery cell and can yield insights into the prevalent degradation mechanisms and in combinations with, e.g., ICA and DVA analysis contribute to revealing the underlying ageing modes and mechanisms during LIB ageing [17,19,20]. In this case, the ageing mechanism occurring when cycling at 5 °C can lead to a safety hazard, that is of extreme importance to reveal if that occurred in a real-life application. A detailed discussion of ageing and degradation mechanisms is outside the scope of this work.

## 4. Conclusions

Within this work, we presented a comprehensive collection of entropy profiles for a total of 14 common commercially available battery cells. The presented data provides valuable insights into the batteries’ thermal characteristics with respect to the reversible heat evolution. The results clearly show that reversible heat can significantly contribute to the overall heat generation in a battery during operation. Consequently, it is in general not enough to only consider heating effects related to irreversible heat such as resistance and overpotentials to understand and describe a Li-ion cell’s accurate temperature profile during charge or discharge.

A direct comparison based on battery chemistry illustrates significant differences between individual electrode chemistries and their combinations into full cells. By looking into different ways of quantifying the entropic contributions, we clearly illustrate that a simple average entropy value across the entire SoC window is not enough to appropriately characterise the reversible heat evolution. Even average entropy values close to zero do not necessarily imply negligible reversible heat contributions to the total heat generation in the cell. Detailed knowledge of entropy, especially its variation with SoC is crucial for the development of appropriate thermal models and thermal management strategies during battery operation in modules and systems. Significantly different heating and cooling rates might be necessary depending on the selected SoC window. This is particularly relevant for LCO and graphite-based electrodes, where significant heating and cooling effects are expected across large SoC ranges. Monitoring the entropy profile of a cell during ageing at different temperatures showed significant shifts also in the cell’s thermal balance, clearly illustrating the need for updated thermal management strategies upon cell ageing.

We hope that the provided data will contribute to the further improvement of both, thermal battery modelling and the development of appropriate thermal management strategies for battery cells, modules and systems.

## Figures and Tables

**Table 2:** Summary of characteristic entropy features by electrode chemistries and comparison to previously reported entropy data for similar chemistries.

Chemistry	Cell Labels (This Work)	Features and Comments	References
LTO	Kokam LTO, Toshiba SciB	Small overall contribution and little SoC variation	Half-cell: [48,49] Full cell: [26]
Graphite	LG JP3, LG E58C, XALT 31 HE, Panasonic UR, Panasonic NCR, SAFT VL6P, SAFT VL30PFe, Melasta 8C, Melasta 10C, Samsung SDI, GPB ICR10440	Characteristic entropy profile following intercalation stages of Li into graphite (see text)	Full cell: [25,46,55,60,61] Half cell: [24,26,44,46,47]
Hard Carbon	Enerdel	Rather featureless, overall low magnitude values with very little variation with SoC	Full cell: [25] Half cell: [44]
NMC	LG JP3, LG E58C, XALT 31 HE, Panasonic UR, Enerdel	Low magnitude entropy, little SoC variation; detailed half-cell entropy measurements necessary to establish differences between different NMC compositions	Full cell: [26,46,51,52,53,60] Half cell: [46,52]
NCA	Panasonic NCR, SAFT VL6P	Low magnitude entropy, little SoC variation	Full cell: [57,58]
LFP	SAFT VL30PFe	Low magnitude entropy, little SoC variation	Full cell: [55,56,61] Half cell: [56]
LCO	Melasta 8C, Melasta 10C, Samsung SDI	Large negative entropy across almost the entire SoC window, except for pronounced peak at around 70–90% SoC (monoclinic-hexagonal phase transition)	Full cell: [26,44] Half cell: [25]
LMO	GPB ICR10440 (part of cathode mix in), Kokam LTO, Toshiba SciB	Low magnitude entropy with characteristic features: maximum around *x* = 0.65, minimum around *x* = 0.55 (*x* in Li_x_Mn_2_O_4_)	Half or full cell: [26,49,54,59]

## Data Availability

The original data presented in the study are included in the article’s Supplementary Material; further inquiries can be directed to the corresponding author/s. This includes: .csv of entropy profiles.

## Supplementary Materials

The following supporting information can be downloaded at: https://www.mdpi.com/article/10.3390/e27040364/s1.
